# A reporting framework for describing and a typology for categorizing and analyzing the designs of health care pay for performance schemes

**DOI:** 10.1186/s12913-018-3479-x

**Published:** 2018-09-04

**Authors:** Yewande Kofoworola Ogundeji, Trevor A. Sheldon, Alan Maynard

**Affiliations:** 1 Health Strategy and Delivery Foundation (HSDF), 1980 Wikki Spring Street Maitama, Abuja, Nigeria; 2 Department of Health Sciences and Hull York Medical School, University of York, Heslington, York, UK

**Keywords:** Performance for performance (P4P), Typology, Design, Heterogeneity, Behaviour, Reporting

## Abstract

**Background:**

Pay for Performance (P4P) has increasingly being adopted in different countries as a provider payment mechanism to improve health system performance. Evaluations of pay for performance (P4P) schemes across several countries show significant variation in effectiveness, which may be explained by differences in design. There is however no reliable framework to structure the reporting of the design or a typology to help analyse and interpret results of P4P schemes. This paper reports the development of a reporting framework and a typology of P4P schemes.

**Methods:**

P4P design features were identified from literature and then explored using relevant theories from behavioural and economic science. These design features were then combined with the help of multidimensional tables to produce a reporting framework and a typology which was tested using 74 P4P studies. The inter-rater reliability of the typology was assessed using Fleiss’ Kappa.

**Results:**

A Healthcare Incentive Scheme Reporting Framework (HISReF) was developed consisting of nine design features. This was collapsed into a typology consisting of 4 items/design features. There was good inter-rater reliability on all the four items on the typology (kappa > 0.7).

**Conclusion:**

The HISReF provides an important first step towards establishing a common language in which intervention designers can clearly specify the content of P4P designs. Our typology may be used to aid evidence synthesis and interpretation of results of P4P schemes.

**Electronic supplementary material:**

The online version of this article (10.1186/s12913-018-3479-x) contains supplementary material, which is available to authorized users.

## Background

Pay for performance (P4P) in healthcare has been adopted in many countries across to aid improvements in health service delivery across a range of clinical areas [1]. It is important that we learn from the experiences of these schemes when deciding if such schemes are useful and cost effective in promoting improved quality of and access to care. There have been several evaluations and reviews of evaluations of P4P schemes and these show significant variation in effect, and it is difficult to make sense of this evidence due to heterogeneity in design, implementation, and context [2–5]. Too often, P4P schemes do not make clear the theoretical basis and justifications for the designs of the schemes. Similarly, evaluations do not relate the findings to the features of the programmes under scrutiny, even though there is a range of theory from behavioural science and economics that can be used to understand better how people respond to incentives.

A few researchers have considered some of the design features of P4P to see how they relate to its effectiveness [6–8], but these have used somewhat ad hoc approaches and there are no studies in the literature combining design features of P4P in a standardised and reliable framework which can be used to explore this variation in systematic way. To sensibly describe, evaluate, and compare P4P schemes, there is a need for a standardised and theoretically informed reporting framework and a way to categorise schemes in a common typology.

This paper contributes to this area by (a) developing a reporting tool for the design features of P4P schemes and (b) constructing, refining and testing the reliability of a typology which can be used to compare and analyse P4P schemes. Developing a typology is particularly important because the design variables of P4P schemes are not mutually exclusive and some of them work synergistically with others or completely nullified by others.

## Methods

We developed the reporting framework and the typology following the ‘constructed type’ method of McKinney (1966) [9, 10] because of the importance of applying it to empirical cases. This method involved five major steps (the first three of which were used to develop the reporting template):

i) Identification from the literature, of design features potentially for inclusion in the typology
ii) Identification and exploration of behavioural science and economics theories which may indicate the relevance of these design features to changing health service behaviour
iii) Combining the design features in a multidimensional space: this involved defining standard criteria for design features identified and combining them in a multidimensional space. This resulted in an original typology which we present as a standardised template for the reporting of characteristics of P4P schemes.
iv) Piloting the typology: The functionality of the P4P typology was tested against a set of pre-defined criteria [11–13]: (1) Relevance: all the core components considered, (2) Manageability and ease of use: not cumbersome with only a few types, (3) Mutual exclusivity: only one type for each P4P program, and (4) Comprehensiveness: whether all the empirical P4P programs could be categorized.
v) Refining/reducing the typology: We reduced the typology using three methods [11, 12]: (a) dichotomization of variables, by merging any variables within design features so that there are just two categories; (b) pragmatic reduction, which involves combining or compressing design features with the same underlying theory or concept [13–15]; (c) rescaling, which involves the removal of less relevant features from the typology.

Following the development and refinement of the typology, we then undertook an assessment of the reliability of the categorisation of schemes using the P4P typology tool by exploring the extent to which raters independently assessing the same papers describing the scheme agreed on their classification. We used Fleiss’ Kappa to assess the inter-rater reliability of all the items on the typology as a P4P categorisation tool [16, 17]. This involved raters/users applying the P4P typology to a sample of reports of P4P studies. We aimed for five independent assessments for each study report. We estimated the sample size for the number of reports of P4P to be assessed based on the probability of detecting a statistically significant kappa (the difference between the overall and chance agreement P_a_-P_e_) with a confidence interval of a desired width as suggested by Sim and Wright [18] and Gwet [19] (see Table 1). In total, 12 volunteer raters used the typology to categorise between 5 and 6 P4P reports. A kappa value of 0.9 (30% relative error) was selected based on a trade-off between precision and a reasonable number of P4P reports to avoiding burdening the raters. This meant the raters had to apply the typology on a minimum of 14 P4P reports. The rater population consisted of five PhD students, four Masters students, and three Masters degree level health service researchers. Four of the raters had between zero to one year of research experience, seven raters had between two to four years of research experience, and one rater had over 5 years of research experience. Only three of the raters had previous experience of P4P schemes in healthcare. A manual was developed to train the volunteer raters which included clear and concise decision rules (with examples) to accompany the guidance for applying the tool to reports of P4P schemes. Volunteer raters were trained face to face or over skype on how to use the typology to categorize P4P schemes. The raters assigned their classification independently of each other using all four items on the typology. All analysis was done on Stata version 12.

**Table 1:** Number of P4P reports needed to estimate kappa

*Pa* − *Pe*	Relative Error		
	20%	30%	40%
0.1	2500	1111	625
0.2	625	278	156
0.3	278	123	69
0.4	156	69	39
0.5	100	44	25
0.6	69	31	17
0.7	51	23	13
0.8	39	17	10
0.9	31	14	8
1.0	25	11	6

## Results

### Identification of design features and theories

The design features we identified from literature include: who receives the incentives, type of incentives, type of payment, size of incentives, method of payment, performance measure, payment mechanism, the time lag between the measurement of performance and payment of the incentive, the domain of performance measured (process, structure, outcome). We then examined these design features considering relevant theories and concepts from behavioural science and economics literature (see Additional file 1: Tables S1 to S3).

### Design features and the relevant theories

#### Who receives the incentives?

P4P schemes involve payment of financial incentives to one or more of: individual health professionals and groups (clinical teams, health institutions) [20–22]. Organizations/groups are capable of setting up good management structures that could be strong enough to elicit a change in behaviour. For example, incentives paid to groups could be used to purchase equipment or hire additional staff, which could lead to improvements in quality and performance [23–27]. This argument is in line with organisation theory which proposes that payment of incentives to groups rather than individuals are more likely to have desired effects because organisations are capable of promoting behaviour change in employees through a wide range of strategies e.g. better structures, improved supervision, enacting stricter guidelines and policies [28]. Although, this is dependent on the quality of managerial or organisational effectiveness and control.

The argument for paying directly to individual health care professionals as opposed to groups or institutions is informed by the ‘free rider’ problem [29–32]. This suggests that individuals are more likely to undersupply the service being incentivised when they share responsibility of providing that service because they might feel that the payment might be shared equally rather than based on individual contributions. Therefore, there is less incentive to try to perform better because as an individual, one can ‘get away with’ not changing behaviour and still receive the incentive. Furthermore, some researchers suggest that paying individual health professionals could create competition among the individual providers, so producing adverse consequences such as hoarding of knowledge and skills, thereby undermining the promotion of team based care, which is believed to be important to improving the quality of care [29].

#### Form of incentive: fines or bonuses (loss aversion theory)

There are two forms of financial incentive used in P4P schemes: fines and bonuses. Kahneman and Tversky developed The Loss Aversion Theory, which explains the tendency for people to prefer to avoid losses compared to acquiring gains. Adam Smith wrote, “*Pain... is in almost all cases a more pungent sensation than the opposite and correspondent pleasure. The one almost always depresses us much more below the ordinary or what might be called the natural state of our happiness, than the other ever raises us above it*” (Smith, quoted in Maynard, 2012, p.8) [33]. From this perspective, fines are more likely to motivate behavioural change than bonuses. In addition, P4P schemes, which use fines, might be more sustainable compared to P4P programmes that only use bonuses because they could be less costly [34]. The implication of this in P4P in health care is that practitioners will be more inclined to change behaviour or increase performance if they think they might lose something rather than get a bonus. However, bonuses are still the most common form of incentives used in P4P programmes in healthcare or a combination of bonuses and fines. This may be because fines can lead to a loss of intrinsic motivation, aggravating clinicians who have altruistic purposes and who might feel they are not being appreciated for their work [35–37] Fines are also harder to administer and to handle from an accounting perspective, particularly in weaker health systems [38].

#### Type of incentive: monetary or non-monetary (justifiability, evaluability, and expectancy theories)

Evaluability theory suggests that some non-monetary incentives are more difficult to value monetarily and may be more desirable as a result. For example, an award in recognition of performance that provides an all-expenses paid holiday to an exotic island is likely to be considered a pleasurable experience. These positive attributes are difficult to ‘put a price on’ and thus may be ascribed a higher value than the cash equivalent. Behaviour change then becomes an effective way of acquiring something that someone could not normally justify purchasing with their own money [39, 40]. Crifo and Diaye found that if agents are continually rewarded with money there is the possibility of reward inflation i.e. the agents get used to the incentives and so might no longer be as motivated by it to change behaviour [41]. Despite this, non-monetary incentives are rarely used in P4P schemes.

A contrary view would be supported by expectancy theory [42], which suggests that: “*individuals act to maximize expected satisfaction with outcomes”*. The theory assumes that individuals’ motivation to work is dependent on two factors: (1) the expectancy about the relationship between effort and a particular outcome and (2) the valence (attractiveness) of the outcome. These two factors are believed to create the motivation that will lead to individuals changing their behaviour towards achieving the desired outcome. Vroom argued that money has valence because it is effective in acquiring things desired by individuals such as material goods of their choice [42]. Therefore, money might be more effective in driving behavioural change compared to non-monetary incentives. This might be particularly true for individuals whose salaries are barely sufficient. In such cases, money might be a more effective driver of behaviour change than non-monetary incentives. Since people can choose how to use monetary incentives, this might be more effective than non-monetary incentives that might be of value to some agents within the same P4P scheme [43]. Furnham and Argyle further argue that money has symbolic value due to its perceived relationship to prestige, status, and other factors [43]. Monetary incentives may have higher valence than non-monetary incentives, depending on the relative payment schedules.

#### Size of incentives (the target income hypothesis)

The most common form of description of size of incentive is the amount of money relative to the clinicians’ salary, usual budget of the health institution, or anticipated payment regarding the health service(s) in question. Other P4P programs simply report the size of incentive in absolute terms as the actual amount earned.

Hahn suggested that the effect of an incentive might be influenced by its size compared to the usual salary, budget, or anticipated payment [44]. Incentives might be too small compared to the usual salary, to induce significant change even when the objectives are measured accurately and fairly evaluated. As the size of the incentives (fines or bonuses) increase, everything else being equal, people may be motivated to work harder to reach the set targets. Though the relationship is likely to demonstrate diminishing marginal returns; after a certain point, increasing the size of incentive might not bring about the required behaviour change, leading to a waste of resources [45]. So, attaching too large an incentive might result in paying more than necessary to bring about the desired behavioural change. The size of incentive also raises the question of cost-effectiveness of P4P schemes, as money spent on the incentive might not be justified by the potential benefits in patient outcomes resulting from behaviour change.

When assessing the size of an incentive in a report of a P4P scheme, therefore, it is best to calculate its value relative to the clinicians’ usual salary/reimbursement. There are no set cut-offs in theory as to what size of incentive is adequate to change behaviour, so we suggest arbitrary cut-offs guided by some empirical evidence. The size of incentives in P4P schemes in healthcare tend to range from 0.5% to up to 100% increase in individual salary or institution budget. Studies indicate that most P4P initiatives with less than 5% increase/decrease in payment had no statistically significant effect on the performance indicator compared to P4P schemes with above 5% in salary or budget [34, 46–48]. For the purpose of developing the P4P typology, we proposed 3 categories of size of incentive (relative to usual reimbursement) namely: small (< 5%), medium (5–10%), and large (> 10%).

There is some evidence that “*physicians have a desired income that they want to achieve whenever their actual income is below that income*” [49]. This is commonly referred to as the Target Income Hypothesis and if valid, it means that increasing the size of incentive would result in an increase in performance only until the clinicians reach their target income after which, increasing the size of incentive may not increase it any further and indeed may reduce performance. Desquins and colleagues [50] found that 80% of physicians would be willing to perform better to reach a target income, a finding supported by other researchers [51, 52]. Those developing P4P schemes, therefore, should have an idea of the average target income of the clinicians participating in the P4P programmes, for example through surveys [53]. In reality clinicians may use some of the additional revenue not as personal income but to enhance facilities [27]. In addition to the effect of the size of incentive relating to income and target income, its impact is also likely to be influenced by the difficulty of reaching the performance or targets that is required to receive the payment. This could mean that what constitutes an adequate incentive to improve performance or reach a certain target in a certain area of healthcare is likely to differ across contexts (such as high and low income countries).

#### Method of payment (coupled or decoupled from usual reimbursement): mental accounting theory

The method of payment in incentive programmes can be coupled or decoupled from salary or income. For example, increasing the usual salary of £2000 to £2080, compared to making a separate payment of £80. Mental Accounting Theory states that individuals divide their current and future assets into separate, non-transferable portions and will assign different levels of utility of each asset in each group [54]. This predicts that people will value incentives more highly if not coupled with the usual salary [39]. Applying this theory to P4P schemes means that it is likely that individuals would place more value on incentives not coupled with the usual salary compared to incentives coupled with salary (even though they might be the same amount). Decoupling the incentives from usual reimbursement might be administratively more burdensome. It could however be worth the additional cost, if it contributes to the success of the P4P programmes.

#### Payment mechanism (absolute or tiered thresholds): the goal gradient theory

There are two main kinds of payment mechanisms in P4P schemes. The first involves a payment for achieving a fixed absolute target (e.g. 70% of people having their blood pressure measured) and the second involves variable and increasing payments triggered at various tiered targets (e.g. 60, 70, and 80%) or a continuous scale.

Goal Gradient Theory [55] predicts a greater positive behavioural response if there are a series of stepped target thresholds [36]. Therefore an incentive payment made for reaching an absolute threshold or a single target might be less effective in changing behaviour compared to one which increases as performance further improves, because individuals in an incentive programme intensify their efforts as they sense that they are getting closer to their target goal [56].

There is also the risk of loss of interest or motivation when the target goal is achieved (this might explain why some successful P4P programmes seem to reach a plateau or even dip after sometime) where there is just one target [57]. This suggests that having tiered targets or a sliding scale might challenge the clinicians to a continued effort in improving performance. Individuals are more likely to be motivated when the target goals appear to be ‘realistic’. Tiered targets might also be more effective if the final target goal is far from the baseline as it might be viewed as unachievable to the individuals, who may see no reason to try to meet the target, as they are likely to fail. In addition to the risk of not getting any payment, this might also reflect the perceived cost to them of achieving it; the expected benefit might be too low.

#### Performance measure, domain of performance, and time lag: risk aversion theory

Risk Aversion Theory tries to explain the behaviour of individuals when exposed to risk or uncertainty. An individual is less likely to change behaviour or do more work the higher the risk of not getting the expected reward, instead they are more likely to focus on activities where the reward is more certain [58]. In P4P schemes in healthcare, there are several elements of risk or uncertainty of not getting paid the anticipated or desired amount, which could reduce the impact of the scheme.

The riskiness of a scheme may be explained in terms of the following P4P design features:

- The degree to which the target takes into account achievement in absolute terms or relative to how others perform (performance measure: absolute or relative measure)
- The degree to which the person/organisation being incentivised can directly control or influence the performance being measured (domain of performance measured)
- The confidence the provider has of being paid if they do improve performance/achieve the relevant target.

#### Performance measure (absolute and relative measures)

Absolute measure of performance is when an incentive is paid for a level of quality improvement, independent of other providers’ performance (e.g. payment per patient immunized). A relative measure, on the other hand, is when incentive is paid for attaining above a specified rank relative to other providers (e.g. payment to clinicians for exceeding the median or bottom quartile immunisation rate). Relative performance measures create greater uncertainty for health service providers because their achievement depends also on how well others do. Providers may be less motivated to invest in improving performance if they have doubts about their performance relative to others. P4P schemes where absolute performance measures are used are, therefore, more likely to be more effective.

#### Domain of performance (to what extent is it within the control of the provider)

The domain of performance measured may be related to the degree of control the provider has on achieving performance improvement expectations and so the level of perceived risk of not being rewarded. The domains of performance that could be measured include:

- Structure: this involves the resources to deliver care e.g. equipment, IT, human resources, facilities, and materials)
- Process: involves performing routine operations, specific tasks or recommended treatments e.g. periodic cholesterol screening, immunization.
- Intermediate outcomes: Intermediate outcomes are the steps or outcomes between the change in behaviour and the final health outcome (e.g. reduction in cholesterol levels, reduction in blood pressure). If evidence-based, there is likely to be a causal link between achieving the intermediate outcome and improvement in final outcomes (e.g. reduction in heart disease). However, this is not guaranteed as other factors may intervene.
- Final outcomes: these are effects on the quality and length of life and wellbeing of people (e.g. reduction in mortality and morbidity rates).

Changes in structure and process (and to a lesser extent intermediate outcome) domains of performance are often seen as more easily achievable because they are more directly under the control of the healthcare organization or clinician, compared with the final (or intermediate) health outcome measures which are influenced by a variety of other factors. Underachievement of final health outcome targets does not always mean there is a quality problem [59]. For example, if a clinician is to be incentivised based on a reduction in cardiovascular mortality rates, the positive efforts by the clinician may be thwarted by lifestyle choices of the patients (e.g. exercise, diet), adherence to treatment and other (e.g. environmental) factors outside their direct control.

For this reason, P4P interventions that focus on the final health outcome domain of performance might be perceived as higher risk (greater uncertainty in earning the incentive payment despite the efforts of the provider) and so might not be as effective in prompting provider behaviour change as incentives linked to changes in structure and process domains of performance. However the schemes might be less effective and cost-effective because structure and process changes do not necessarily translate into improved health [60].

#### Timing of payment (and frequency of payment)

Timing of incentive payment ranges from monthly to annually. When the time lag between the measurement of performance and payment of incentives is longer it can create some uncertainty, particularly in countries with a track record of or poor administrative infrastructure, corruption and political instability. This uncertainty in payment might reduce the motivation to improve performance. In addition, shorter time lags between payments may indicate smaller more frequent payments, which are more likely to motivate a higher behavioural response in an individual compared to a one-time lump sum incentive [61]. A randomised controlled trial conducted in the USA compared annual payments to quarterly payments of incentives to individual physicians worth $5000 overall for quality improvements in treatments and outcomes of diabetes, cancer screening, and smoking [62]. It found that quarterly performance group performed better but this was because in this arm, they had to present reports every quarter to be approved for the payment of the incentive, which might have contributed to motivating the physicians in this group compared to submitting yearly reports.

Furthermore, individuals often exhibit time preference (or time discounting) where *“happiness now is worth more to me than happiness next year”* [63]. Consequently, individuals perceive incentives received soon after the behavioural change as having more value than the same amount received in the future, (pure time preference). Loewenstein and Prelec [64] also suggest that time lag between measurement of performance and the receipt of the incentives could affect behavioural response. Individuals tend to ask themselves; is there anything that I could do now that will bring me immediate rewards instead of what I could do now that would reward me in a years’ time? Consequently, P4P designs with short time lags between provision of care and receipt of incentive might be expected to produce greater behavioural response.

Some P4P schemes may take months or even a year or more to collect and validate performance data. People might be relatively motivated to change their behaviour even if the payment is a year away (after measurements of performance) for very large incentives, which implies that these design features might interact with each other to influence the impact of the scheme. This is another advantage of developing a typology, as each type (category) will be a unique combination of the dimensions of the design features of P4P.

Previous studies have suggested that monthly, bi-monthly, or quarterly payments constitute shorter time lags, while payments after 4 months constitute a long time lag [6, 7, 65, 66]. For the purpose of categorisation in this typology, monthly to quarterly payments were considered as short time lags, whereas, payments made after 4 months were considered long time lags.

#### Reliability of measurement of performance

Similar to the timing of payment, the reliability of measurement of performance could also affect the confidence that the health service provider has in being paid if they do achieve the relevant target. Clinicians are likely to perceive the potential of earning the incentive as more uncertain if the tool for measuring performance is not reliable. Providers will most likely not make great efforts to change their behaviour if they might think that the measurement tool might not accurately reflect the consequent improvement in performance. It is difficult to judge reliability from reports of schemes as it depends partly on the perceptions of the providers in the particular context, which are not commonly reported in P4P evaluations. This should be explored as part of the implementation context when designing a scheme.

### A standardised template for the reporting of characteristics of P4P schemes

Table 2 below lays out the nine key design features of P4P schemes that we have found from the theoretical and empirical literature as likely to affect the impact of the scheme on changing provider behaviour. When considered together, they constitute a reporting framework or template – the Healthcare Incentives Reporting Framework (HISReF). In order to increase the transparency and consistency of reporting of P4P schemes and their evaluations, we recommend that authors provide information on each of these nine features, over and above other details.

**Table 2:** Healthcare Incentives Reporting Framework (HISReF) - a template for reporting standard features of P4P schemes

Core design features	Variables	Description
Who receives the incentives?	Individuals	Incentive is paid to an individual health care provider e.g. physician
	Groups	Incentive is paid to a group and individual clinicians might not benefit from the incentive directly e.g. hospital trust, clinical team, general physician (GP) practice, NGO, levels of government, faith based organizations
Type of incentive	Bonuses	Incentive is in the form of increase in payments, bonus, gifts, peer recognition etc.
	Fines	Negative incentives in the form of reduction in expected payments, penalty, punishment etc.
Type of payment	Monetary	Incentive in form of money
	Non-Monetary	Incentives in the form of material things or tangible gifts
Size of incentive	Large	Monetary or non-monetary reward or fine- > 10% of salary, budget, or anticipated payment
	Medium	Monetary or non-monetary reward or fine 5–10% of salary, budget, or anticipated payment
	Small	Monetary or non-monetary reward or fine < 5% of salary, budget, or anticipated payment
Payment mechanism	Absolute	Incentives are paid as a single payment for an absolute increase in performance for example, an 80% increase in performance.
	Tiered thresholds	Incentives are paid for a series of target thresholds to meet for example paying increasing incentives for achieving a 65%, an 80%, and a 90% performance threshold.
Method of payment	Coupled	Incentives paid are coupled with usual reimbursement e.g. an incentive in form of an increase in salary.
	Decoupled	Incentives are paid separately from the usual reimbursement.
Performance measure/payment scale	Absolute measure	Incentive is paid for improvement in performance or behaviour change not dependent on other providers e.g. incentive paid per patient immunized
	Relative measure	Incentive is paid for attaining a level of performance relative to other providers e.g. incentives paid to clinicians or hospitals above the median performance
Domain of performance measured	Within clinicians control	Incentive payments are based on process and structural outcomes e.g. having the right equipment, the number of children immunized, routine measurement of blood pressure of patients every month
	Out of clinicians control	Payment of incentives to health providers for ultimate health outcomes e.g. reduction in mortality rates from a specific disease
Time lag	Short	Payment of incentives four months or less after measurement of performance
	Long	Payment of incentives more than four months after measurement of performance

### Combining the design features in a multidimensional space: development of the typology

In order to produce a typology, these features need to be combined in a multidimensional space and doing this with the number of design features identified would result in 108 possible types, too many to be useful as an analytical tool. So we reduced these to a smaller number that would be usable, but still sufficiently informative to work as analytical tool.

### Reducing the typology

Each of the nine design features identified in Table 2 had two categories apart from ‘size of incentive’ with 3 categories: small, medium, and large. We dichotomized this further by merging the medium and large categories, because theory suggests that medium and large incentives are more likely to have similar effects compared to small and medium incentives. This reduced the typology to around 81 unique types/cells.

This was followed with a pragmatic reduction that involved merging design features with the same underlying theory. Three design features shared Risk Aversion theory: timing of payment, domain of performance measured, and performance measure. These were collapsed into one conceptual variable called the ‘Perceived Risk of not earning the incentive’ (Risk), with two categories: low risk and high risk. In the ‘low risk’ category, clinicians perceive the incentivised entity as a performance target that is achievable and there is little or no risk of not getting paid the incentives. In the ‘high risk’ category, there is no guarantee of payment because the relative performance depends on that of others, which introduces an element of risk [58]. Table 3 shows the new conceptual (collapsed) dichotomous variable, ‘perceived risk of not earning the incentive’ (Risk): low risk and high risk. Individuals who perceive the risk or uncertainty associated with earning the incentive as low are more likely to change behaviour because there is a higher guarantee about earning the incentive compared to when individuals perceive the risk associated with earning the incentive as high.

**Table 3:** Collapsed variables to form a conceptual variable ‘Risk’

(Risk) Collapsed variables	Categories of new variable	
	Low risk	High risk
Performance measure	Absolute: incentive is paid for quality improvement not dependent on other providers e.g. incentive paid per patient immunized	Relative: incentive is paid for attaining a specific rank relative to other providers e.g. incentives paid to clinicians or hospitals in top 2 performing quartiles
Domain of performance measured	Within clinicians control: incentive payments are based on process and structural outcomes e.g. number of children immunized, routine measurement of blood pressure of patients every month	Not within clinicians control: payment of incentives to health providers for health outcomes e.g. reduction in blood pressure of patients or reduction in mortality rates from a specific disease
Time lag	Short time lag: Payment of incentives immediately after measurement of performance) or four months or less.	Long time lag: Payment of incentives more than 4 months after measurement of performance

To ensure that the typology is mutually exclusive (no P4P schemes falls into more than one type) and to ensure that as many P4P schemes as possible can be categorized (despite poor reporting of features in some studies), we set a decision rule that: a P4P scheme is categorized as low risk if it has two or more of: short time lag, domain of performance within clinicians’ control, and absolute performance measure. A P4P scheme is categorized as high risk if it has two or more of: long time lag, domain of performance out of clinicians’ control, and relative performance measure. So whilst these features should be reported separately in the HISReF, they were collapsed into one for the typology. This pragmatic reduction method resulted in 49 types; but this was still too many to be useful in analysis.

Finally, we rescaled the typology by removing the three least relevant or useful design features [11, 12], as judged by their degree of variability within the empirical P4P cases in literature. They were: kind of incentive (monetary and non-monetary) because in reported P4P schemes the main form of incentive used was money; method of payment (coupled and decoupled) as payment is mainly decoupled from usual payments; and mechanism of payment (absolute and tiered threshold), (monetary incentive) as the mechanism of payment for a majority of the schemes was absolute. These features are still important in the designing and reporting P4P schemes, however, for the purpose of the development of the typology, these features would not contribute significantly to the analytical and theory-testing functions of the typology. This reduction resulted in a final typology of four design features, each consisting of two categories and a more manageable typology of 16 possible types (Table 4):

- Who to incentivise (individuals or groups)
- Type of incentive (fines or bonuses)
- Size of incentives (small or large)
- Perceived Risk/uncertainty of payment (low risk or high risk)

**Table 4:** P4P Typology

Type	Who received the incentive	Type of incentive	Size of incentive	Perceived risk of not earning the incentive (RISK)
1	Groups	Fines	Large	Low
2	Groups	Bonuses	Large	Low
3	Groups	Fines	Small	Low
4	Groups	Bonuses	Small	Low
5	Groups	Fines	Large	High
6	Groups	Bonuses	Large	High
7	Individuals	Fines	Large	Low
8	Individuals	Bonuses	Large	Low
9	Groups	Bonuses	Small	High
10	Groups	Fines	Small	High
11	Individuals	Fines	Small	Low
12	Individuals	Bonuses	Small	Low
13	Individuals	Fines	Small	High
14	Individuals	Bonuses	Large	High
15	Individuals	Bonuses	Small	High
16	Individuals	Fines	Large	High

### Piloting the typology

The relevance had already been demonstrated through the process of developing the typology, which involved thorough consideration of relevant theories and literature applicable to design variables of P4P. Similarly, manageability was achieved through reduction of the typology to a few types to facilitate its use in analyses. Schemes with a combination of bonuses and fines were categorised alongside those with only fines. This follows the rationale that individuals are still likely to manifest ‘loss aversion’ as long as there is an element of fine or penalty and whether there is the potential to earn bonuses or not is not likely to deter the risk averse behaviour [35]. We also redefined the criteria for categorization of payment of incentives to groups to include instances where individuals may or may not benefit from the group payments. This is because when incentives are paid to groups as opposed to individual clinicians, one of the ways a management system could motivate behaviour change within the organisation is to provide individuals an opportunity to earn from the incentives received by the group. Where schemes had a mixture of process and outcome measures we categorised them according to the predominant measures. For example, P4P schemes with four outcome measures and 20 process measures were categorized as mostly under the clinicians’ control, since there are more processes than outcomes, as opposed to ten outcome measures and two process measures, which will be categorised as mostly out of the clinicians’ control. In addition, in the unlikely case where there are equal number of processes and outcomes, the outcome measures are likely to outweigh the process measures. The resulting final version of the typology is shown in Table 5.

**Table 5:** Final version of the P4P typology

ITEM 1: Who received the incentive? Did Individuals or Groups receive the incentive?	
Criteria for judging Individuals	• If the incentives are paid directly to individual health workers/clinicians/doctors only • If individual health worker/clinician/doctor’s income is supplemented as a result of the incentive (e.g. reflected in the rise of personal income) only
Criteria for judging Groups (including schemes where individuals and groups are paid bonuses)	If the incentive is paid to a group or an organization in which individual clinicians may or may not benefit from the incentive directly Groups include any of the following • Hospital • Clinical team • General physician (GP) practice • NGO • Levels of government • Faith based organizations
ITEM 2: Type of incentive Was the incentive in the form of Fines or Bonuses?	
Criteria for judging Fines	If the incentive is negative in the form of reduction in expected payments, penalty, punishment etc. In some cases, bonuses may or may not be paid.
Criteria for judging Bonuses	If incentive is in the form of increase in payments, bonus, gifts etc. with NO fines levied
ITEM 3: Size of the incentive Was the size of the incentive small or large?	
Criteria for judging Small	If the incentive in the P4P program is smaller than 5% of any one of the following: • Salary of individual clinician/health worker/doctor • Anticipated payments (to the health facility/hospital/clinical team) such as budgets (total budget or budget for the particular intervention in question), fee for service (FFS) and capitation
Criteria for judging Large	If the incentive in the P4P program is 5% and above of any one of the following: • Salary of individual clinician/health worker/doctor • Anticipated payments (to the health facility/hospital/clinical team) such as budgets (total budget or budget for the particular intervention in question), fee for service (FFS) and capitation
ITEM 4: Perceived Risk of not earning the incentive: High risk or low risk? (based on: Timing of payment after achieving targets (time lag), Domain of performance measure, and Performance measure (payment scale)	
Criteria for judging High risk	If the P4P program has 2 or more of the following features • If incentive payment (or penalty) is made after 4 months after measurement and confirmation of performance (long time lag) • If the domain of performance measure was mostly out of clinicians control • If the perofmance measure (payment scale) is a relative measure
Criteria for judging Low risk	If the P4P program has 2 or more of the following features • If incentive payment (or penalty) is made before or at 4 months after measurement and confirmation of performance (short time lag) • If the domain of performance measure was mostly within the clinicians’ control • If the performance measure (payment scale) is an absolute measure Note: It is possible to judge the risk of the program if one feature is missing/unclear. For example, if the time lag for payment is short and the domain of performance measure was mostly within the clinicians’ control. We can judge from this information that the risk is low even when there is little or no information about the performance measure
Timing of payment after achieving targets (time lag): was it short or long?	
Criteria for judging short	If incentive payment (or penalty) is received not more than 4 months after measurement and confirmation of performance
Criteria for judging long	If incentive payment (or penalty) is received more than 4 months after measurement and confirmation of performance
Domain of performance measured Was the domain of performance measured within clinicians’ control or out of clinicians’ control?	
Criteria for judging within clinicians control	If incentive payments to health service providers are mostly/only based on processes and structures e.g. number of children immunized, routine measurement of blood pressure of patients every month, number of referrals made, rate of cancer screening
Criteria for judging out of clinicians control	If incentive payments to health service providers depend on achieving a change in health outcomes e.g. reduction in mortality rates from a specific disease, blood pressure reduction, patient experience etc. Note: sometimes, incentive programs contain a mixture of processes and outcomes. However, one category out of the two is usually predominant. For example a program with 6 process measures and 2 outcome measures. You will have to judge what category it falls into by deciding which category is predominant and for this example, the incentive program falls within the clinicians control because the process measures are predominantly more than the outcome measures.
Performance measure (payment scale) Absolute or relative measure?	
Criteria for judging Absolute measure	If incentive is paid (fine levied) to the health service provider that based on their performance, not relative to how other health providers perform. For example, • Improvement in performance typically improvement from some baseline measure, using performance score/ performance points achieved • Achieving performance at/above a predetermined target • e.g. incentive paid per patient immunized, or 70% improvement from baseline
Criteria for judging Relative measure	If incentive payment is based on the performance of health service providers, relative to that of other providers. For example, • If bonuses are paid for to health service providers in a specific performance rank e.g. the providers above the top quartile of performance. • And/or • If fines are levied on health service providers in certain ranks usually the bottom ranks e.g. the providers below the lower quartile of performance

This typology was then applied again to all descriptions of P4P schemes from evaluated studies identified from reviews shown earlier in Additional file 1: Table S2. In total, we applied the typology to characterise 73 P4P schemes into mutually exclusive categories using the design features (see Additional file 1: Table S4). Table 6 below shows results of application of the typology on a set of P4P schemes identified from the review by Eijkenaar [14] (results of application of the typology on other P4P schemes identified from other reviews are shown in Additional file 1: Table S4). Whilst we were able to categorize the P4P schemes using all items of the typology, the size of incentive was the most difficult to categorise because studies often used vague terms such as ‘modest’ or ‘small’, without providing absolute amounts or sizes relative to the usual clinician income or hospital budget. However, we were still able to categorize 46 schemes: 32 schemes had large incentive sizes and 14 schemes had small incentive sizes, which to a certain extent suggest that there was a good distribution between the size of incentives across the programs, demonstrating the usefulness and exhaustiveness of the typology.

**Table 6:** Results of applying the typology to P4P schemes identified from the review by Eijkenaar et al. [59]

P4P schemes	Who receives the incentive	Type of incentive	Size of incentive	Time lag	Performance measured	Domain measured	Risk
Advancing quality (AQ) UK	Groups	Bonuses	Small	Short: 2/3 months lag	Relative	Mostly within Physicians control (2 final outcomes and 26 processes)	High
Clalit Israel	Groups	Bonuses	Dependent on budget savings	Long: Annually	Absolute	Mostly within Physicians control (10 processes and 8 intermediate outcomes)	Low
Clinical Practice Improvement Pay (CPIP) Australia, Queensland	Groups	Bonuses	Large	Short: 3 month lag	Absolute	Within physicians control (12 structures and 7 processes)	Low
ERGOV Germany	Groups	Fines	Depend on other hospitals	Short: 4 month lag	Relative	Not completely within the physicians control (Final outcome)	High
MACCABI Israel	Groups	Bonuses	Size not reported	Long: Annually	Absolute	Mostly within Physicians control (12 processes and 5 intermediate outcomes)	Low
National Health Insurance P4P (NHI-P4P) Taiwan	Groups	Bonuses	Large	Short: Monthly	Relative	12 structures, 3 final outcomes, and 2 intermediate outcomes	High
Primary care P4P (PC-P4P) Netherlands Primary Care	Groups	Bonuses	Large	Long: Annually	Relative	Within physicians control (31 processes)	High
Renewal Models (PCRM) Canada Ontario	Groups	Bonuses	Small	Long: Annually	Absolute	Within physicians control (12 processes)	Low
Physician Integrated Network (PIN) Canada Manitoba	Groups	Bonuses	Maximum payment unknown	Short: Immediately after performance measure	Absolute	Within physicians control (only processes)	Low
Practice Incentive Program (PIP) Australia	Groups	Bonuses	Size not reported relative to income	Short: Semi-annually and annually	Absolute	Within physicians control (only structures and processes)	Low
Performance management Program (PMP) New Zealand	Groups	Bonuses	Small	Long: Semi-annually and annually	Absolute	Within physicians control (8 processes)	Low
Program of quality Improvement (PQI) Argentina	Groups	Bonuses	Large	Long: Annually	Absolute	Mostly within physicians control (16 processes, 7 structures and 3 outcomes)	Low
Quality and Outcomes Framework (QOF) UK	Groups	Bonuses	Large	Long: Annually	Absolute	Mostly within physicians control (85% processes)	Low

### Inter-rater reliability (kappa) of each item on the P4P typology

Kappa estimates for each of the four items on the typology are shown in Table 7. Kappa values for who receives the incentive and type of incentive were high at > 0.9. Kappa for size of the incentive and perceived risk of not earning the incentive were lower at 0.72 and 0.71 respectively, though still considered good inter-rater agreement [16, 67]. Sources of disagreements between the raters were random and not specific to any rater. The sources of disagreement in the third and fourth item (size of incentive and perceived risk of not earning the incentive) reflected subjective rater judgement or lack of clarity from study reports. Details of studies assessed, rater characteristics and sources of disagreement between raters are found in Additional file 1: Tables S5 to S8.

**Table 7:** Kappa values for each item on the P4P typology

Items on the typology	Kappa	Z	Prob > Z
Item 1(who receives the incentive: individuals or groups)	0.9510	12.40	0.0000
Item 2 (type of incentive: fines or bonuses)	0.9145	11.92	0.0000
Item 3 (size of incentive: small or large)	0.7157	9.33	0.0000
Item 4 (perceived risk of not earning the incentive: low or high)	0.7059	9.20	0.0000

## Discussion

The reporting framework (HISReF) developed in this study was derived from the empirical and theoretical literature and consisted of nine general features likely affect the effectiveness of a healthcare incentive scheme. From this framework, we then developed a typology by merging and consolidating the design features. The final typology consists of four key design variables: who receives the incentives, type of incentives, size of incentives, and perceived risk of not earning the incentive (a condensed variable consisting three design features: performance measure, time lag between the measurement of performance and payment of the incentive, and the domain of performance measured).

### Limitations

There were three main limitations. There was a trade-off between the typology being manageable and maintaining relevance and utility. Some of the design features explored and discussed (such as method of payment and kind of incentive) whilst included in the reporting framework were removed from the typology and others were collapsed. Thus the typology is not exhaustive and so may not distinguish between schemes with sufficient granularity. Nonetheless, this typology can provide a foundation towards standardised categorizations of current P4P designs in literature.

The second limitation was the problem of poor reporting of P4P scheme evaluations. We chose the best reported studies to test the reliability of the typology and this does not necessarily reflect the reality where most of the P4P designs are not completely reported. Some evaluation studies incompletely reported important design features, despite the potential association between design features and effectiveness of the schemes. This restricted the choice of studies given to the raters for the inter-rater reliability test, which may have led to an over-estimate of the reliability of the typology.

The typology, combines several theories and design features to help describe, categorize, and analyze P4P schemes. However, there are limitations in that the theories explored may not necessarily be applicable to all individuals or cases. For example, in the case of risk aversion theory, providers will vary in their degree of risk aversion or appetite. Similarly, the target income hypothesis relates primarily to physicians’ behaviour and might not necessarily be applicable to other health professionals who are offered performance bonuses in some contexts. In addition, the theoretical models, by assuming at times a simple mechanism of effect on motivation and performance, ignore that they may interact to influence behaviour in complex ways.

The HISReF reporting framework includes a comprehensive range of nine general design features derived from theory and empirical evidence on the likely impact of design features on the effectiveness of incentive schemes in health care. The typology was developed from a subset of these design features and was applied successfully to categorise a number of P4P studies into mutually exclusive categories. It has face validity and strong content validity in that the process of development of the typology was transparent and decisions made were adequately justified and relevant to empirical cases in literature. Overall, all four items on the typology demonstrated good inter-rater reliability; all kappa values were above 0.7. [67, 68, 69]. This implies that if the typology is adopted as a P4P categorisation tool, misclassifications of P4P schemes due to rater error will be minimised. The inter-rater reliability of the size of incentive K = 0.72 and perceived risk of not earning the incentive K = 0.71were moderately lower than the first two items (who receives the incentives and type of incentive) because the latter were typically reported better in the studies, and were easy to identify. This illustrates how important it is that there is better reporting of P4P designs in general and in evaluation studies in particular.

Adoption of the HISReF reporting framework would also be helpful in facilitating effective communication between people who design or adopt, implement or evaluate P4P schemes. It would help provide structured information to P4P designers and developers, so that they understand the possible results of their design choices and possibly help guide their thinking.

The typology should aid analysis and interpretation of the heterogeneous results of the evaluated P4P schemes. The typology now needs to be further developed by applying it to the literature. For example, design variables not included in this typology might be relevant in the near future and added on in a more extensive typology. Though adding more design variables to the current typology might make it a cumbersome framework to be used for analyses and exploration heterogeneity.

The HiSREF and P4P typology were designed to be able to describe, categorize, and analyze whole P4P schemes, however, there are some cases (especially for very large schemes with multiple indicators) where only a few indicators are evaluated at once. The P4P typology is still relevant as it provides a structured way to describe the design features within which these indicators sit and are used. Even if only some indicators are evaluated or design features modified, it is important to understand the whole scheme context as well as the particularities under consideration.

This reporting framework and derived typology of P4P design features provides only one set of tools to understand P4P schemes. Factors over and above design features may affect the impact of schemes [26, 27, 70–72] such as:

- The context in which the P4P scheme is implemented (health systems, increased funding, and complexity)
- How well the program is being piloted: use of baseline measurement, setting of targets, degree of preliminary work done
- Rigour of evaluation (absence or presence of control groups)
- Clinical area of intervention.

## Conclusion

This newly developed reporting framework (HISReF) and the analytic typology derived from it are contributions to understanding the influence design features has on the impact of P4P incentive schemes given the number of schemes being developed across the world. Our research suggests that the reporting framework and typology are ready for use and further development by other researchers, as simple and effective tools to describe and categorise well reported P4P schemes in health care. Their adoption will improve the development of an interpretable evidence base through more structured evidence synthesis and interpretation of results of evaluations of incentive schemes in health care.

## Additional file

Additional file 1: **Table S1.** Search strategy output for Cochrane database. This table details the search strategy employed to identify relevant studies and reviews used in the manuscript. This includes the database searched, years covered, and number of citations. **Table S2.** Summary of identified reviews. This table outlines the relevant reviews and P4P evaluation studies identified from our search strategy, which informed our reporting framework and typology. **Table S3.** Search strategy output for economic theories to inform the P4P typology. This table details the search strategy employed to identify relevant economic theories that were used to construct the P4P typology. This includes the database searched, years covered, and number of citations. **Table S4.** Application of the typology on selected identified P4P schemes. This table outlines the results of applying the P4P typology to categorized identified P4P schemes. **Table S5.** P4P studies used in testing the inter-rater reliability of the P4P typology. This table list out the P4P studies that were selected for the raters to apply the P4P typology. **Table S6.** Rater population. This table describes the rater population i.e. qualifications, research experience, and experience with P4P in healthcare. **Table S7.** Sources of disagreement between raters. This table highlights the items on the P4P typology that were sources of disagreement between he raters. **Table S8.** An example of source of disagreement between raters (risk). This table details text extracts from the sample P4P study and describes the reason for disagreement between raters testing the P4P typology. (DOCX 127 kb)

## Abbreviations

HISReF: Healthcare Incentives Reporting Framework
P4P: Pay for performance
