# Supplementary material for: A reporting framework for describing and a typology for categorizing and analyzing the designs of health care pay for performance schemes
Source: BMC Health Serv Res. 2018 Sep 4;18:686. doi: 10.1186/s12913-018-3479-x (PMC6123918; doi:10.1186/s12913-018-3479-x)
Supplement: Supplementary file 1 — Table S1. Search strategy output for Cochrane database. This table details the search strategy employed to identify relevant studies and reviews used in the manuscript. This includes the database searched, years covered, and number of citations. Table S2. Summary of identified reviews. This table outlines the relevant reviews and P4P evaluation studies identified from our search strategy, which informed our reporting framework and typology. Table S3. Search strategy output for economic theories to inform the P4P typology. This table details the search strategy employed to identify relevant economic theories that were used to construct the P4P typology. This includes the database searched, years covered, and number of citations. Table S4. Application of the typology on selected identified P4P schemes. This table outlines the results of applying the P4P typology to categorized identified P4P schemes. Table S5. P4P studies used in testing the inter-rater reliability of the P4P typology. This table list out the P4P studies that were selected for the raters to apply the P4P typology. Table S6. Rater population. This table describes the rater population i.e. qualifications, research experience, and experience with P4P in healthcare. Table S7. Sources of disagreement between raters. This table highlights the items on the P4P typology that were sources of disagreement between he raters. Table S8. An example of source of disagreement between raters (risk). This table details text extracts from the sample P4P study and describes the reason for disagreement between raters testing the P4P typology. (DOCX 127 kb) [file 12913_2018_3479_MOESM1_ESM.docx]

**Additional file 1**

**Table S1. Search strategy output for Cochrane database**

| **Database** | **Cochrane** |
| --- | --- |
| Host | http://onlinelibrary.wiley.com/cochranelibrary/ |
| Date of search | January 2012-June 2014 last date searched: 26/6/14 |
| Years covered | 1990-2014 no date restrictions |
| Search Strategy | Key word search: Financial incentives, Pay for performance, Performance based financing  There are 20 results from 8524 records for your search on 'financial incentive or pay for performance or performance based financing in Title, Abstract, Keywords in Cochrane Reviews'  There are 12 results from 30299 records for your search on 'financial incentive or pay for performance or performance based financing in Title, Abstract, Keywords in Other Reviews'  There are 3 results from 16096 records for your search on 'financial incentive or pay for performance or performance based financing in Title, Abstract, Keywords in Economic Evaluations' |
| Language restrictions | None |
| Number of citations | 35 |
| Relevant reviews | 8: Huang et al., 2013, Gillam et al., 2012, Reda et al., 2012, Chaix-couturier et al., 2012, Hamilton et al., 2013, Witter et al 2012, Scott et al 2011, Petersen et al 2006, |
| **Database** | **Medline** |
| Host | <http://www.ncbi.nlm.nih.gov/sites/entrez> (Pubmed) |
| Date of search | January 2012-June 2014 last date searched: 26/6/14 |
| Years covered | 1990-June 2014 (no date restrictions) |
| Search Strategy | 1. Search (((((((financial incentive*) OR performance based financing) OR pay for performance) OR paying for performance) OR incentive*) AND Review[ptyp] AND Humans[Mesh] AND English[lang])) AND health |
| Language restrictions | None |
| Number of citations | 1453 |
| Relevant reviews | 12: Van Herck P et al 2010, de Bruin SR, et al 2011, Witter et al 2012, Scott et al 2011, Petersen et al 2006, Eijkenaar 2012, Christianson et al 2008, Reda et al., 2012, Hamilton et al., 2013, Houle et al., 2012, Gillam et al., 2012, Andrew D Oxman and Atle Fretheim, 2009 |

**Table S2. Summary of identified reviews**

| **Reviews** | **P4P evaluation studies** |
| --- | --- |
| 1. OXMAN, A. D. & FRETHEIM, A. 2009a. Can paying for results help to achieve the Millennium Development Goals? A critical review of selected evaluations of results-based financing. *J Evid Based Med,* 2**,** 184-95. 2. CANAVAN, A., TOONEN, J. & ELOVAINIO, R. 2008. Performance Based Financing: An international review of the literature Mauritskade, Amsterdam: KIT Development Policy & Practice 3. CHAIX-COUTURIER, C., DURAND-ZALESKI, I., JOLLY, D. & DURIEUX, P. 2000. Effects of financial incentives on medical practice: results from a systematic review of the literature and methodological issues. *Int J Qual Health Care,* 12**,** 133-42. 4. Christianson JB, Knutson DJ, Mazze RS. Physician pay-for-performance. Implementation and research issues. *J Gen Intern Med.* 2006;21(2):S9-S13. 5. DE BRUIN, S. R., BAAN, C. A. & STRUIJS, J. N. 2011. Pay-for-performance in disease management: A systematic review of the literature. *BMC Health Services Research,* 11. 6. EIJKENAAR, F. 2012. Pay for performance in health care: an international overview of initiatives. *Med Care Res Rev,* 69**,** 251-76. 7. GILLAM, S. J., SIRIWARDENA, A. N. & STEEL, N. 2012. Pay-for-performance in the United Kingdom: impact of the quality and outcomes framework: a systematic review. *Ann Fam Med,* 10**,** 461-8. 8. HAMILTON, F. L., GREAVES, F., MAJEED, A. & MILLETT, C. 2013. Effectiveness of providing financial incentives to healthcare professionals for smoking cessation activities: systematic review. *Tob Control,* 22**,** 3-8. 9. HUANG, J., YIN, S., LIN, Y., JIANG, Q., HE, Y. & DU, L. 2013. Impact of pay-for-performance on management of diabetes: a systematic review. *Journal of evidence-based medicine* 6**,** 173-84. 10. Houle SK, McAlister FA, Jackevicius CA, Chuck AW, Tsuyuki RT. Does performance-based remuneration for individual health care practitioners affect patient care?: a systematic review. *Ann Intern Med.* 2012;157(12):889-899. 11. PETERSEN, L. A., WOODARD, L. D., URECH, T., DAW, C. & SOOKANAN, S. 2006. Does pay-for-performance improve the quality of health care? *Ann Intern Med,* 145**,** 265-72. 12. REDA, A. A., KAPER, J., FIKRELTER, H., SEVERENS, J. L. & VAN SCHAYCK, C. P. 2009. Healthcare financing systems for increasing the use of tobacco dependence treatment. *Cochrane Database Syst Rev,* 15. 13. SCOTT, A., SCHURER, S., JENSEN, P. H. & SIVEY, P. 2009. The effects of an incentive program on quality of care in diabetes management. *Health Econ,* 18**,** 1091-108. 14. VAN HERCK, P., DE SMEDT, D., ANNEMANS, L., REMMEN, R., ROSENTHAL, M. & SERMEUS, W. 2010. Systematic review: Effects, design choices, and context of pay-for-performance in health care. *BMC Health Services Research,* 10**,** 1-13. 15. WITTER, S., FRETHEIM, A., KESSY, F. L. & LINDAHL, A. K. 2012. Paying for performance to improve the delivery of health interventions in low- and middle-income countries. *Cochrane Database Syst Rev,* 15. | 1. An LC, Bluhm JH, Foldes SS, Alesci NL, Klatt CM, Center BA (2008). A randomized trial of a pay-for-performance program targeting clinician referral to a state tobacco quitline. Archives of Internal Medicine; 168(18):1993-1999. 2. Armour BS, Friedman C, Pitts MM, Wike J, Alley L, Etchason J (2004). The influence of year-end bonuses on colorectal cancer screening. Am J Managed Care; 10(9):617-624 3. Ashworth M, Lea R, Gray H, Rowlands G, Gravelle H, Majeed A (2004). How are primary care organizations using financial incentives to influence prescribing? Journal of Public Health; 26(1):48-51. 4. Basinga P, Gertler P, Binagwaho A, Soucat A, SturdyJ, Vermeersch C. (2011). Paying primary health facilities for performance in Rwanda. World Bank, Washington, DC, Policy research working paper 5190. 5. Beaulieu, N. D., & Horrigan, D. R. (2005). Organizational processes and quality. Putting smart money to work for quality improvement. *HSR: Health Services Research, 40,* 1318-1334. 6. Bardach, N. S., J. J. Wang, et al. (2013). "Effect of pay-for-performance incentives on quality of care in small practices with electronic health records: a randomized trial." Jama 310(10): 1051-1059. 7. Bischoff, K., A. Goel, et al. (2013). "The Housestaff Incentive Program: improving the timeliness and quality of discharge summaries by engaging residents in quality improvement." BMJ Qual Saf 22(9): 768-774. 8. Boland, G. W., E. F. Halpern, et al. (2010). "Radiologist report turnaround time: impact of pay-for-performance measures." AJR Am J Roentgenol 195(3): 707-711. 9. Calikoglu, S., R. Murray, et al. (2012). "Hospital pay-for-performance programs in Maryland produced strong results, including reduced hospital-acquired conditions." Health Aff 31(12): 2649-2658. 10. Calvert M, Shankar A, McManus RJ, Lester H, Freemantle N. (2009). Effect of the quality and outcomes framework on diabetes care in the United Kingdom: retrospective cohort study. BMJ; 338:b1870. 11. Campbell S, Reeves D, Kontopantelis E, et al. (2007). Quality of primary care in England with the introduction of pay for performance. N Engl J Med ;357:181e90. 12. Campbell SM, Reeves D, Kontopantelis E, et al. (2009) Effects of pay for performance on the quality of primary care in England. N Engl J Med;361:368e78. 13. Canavan A, Swai G. (2008). Payment for Performance (P4P) Evaluation: Tanzania Country Report for Cordaid Godfrey Swai, National Consultant Width 1. KIT, Amsterdam. 14. Cattaneo A, Borgnolo G, Simon G. (2001). Breastfeeding by objectives. European Journal of Public Health ; 11(4):397-401. 15. Chang FC, Hu TW, Lin M, et al. (2008). Effects of financing smoking cessation outpatient services in Taiwan. Tob Control ;17:183e9. 16. Chee G, His N, Carlson K, Chankova S, Taylor P. (2007). Evaluation of the first five years of GAVI immunization services support funding. Prepared for the GAVI Alliance Behtesda, MD: Abt Associates Inc,. 17. Chen, J. Y., H. Tian, et al. (2010). "The effect of a PPO pay-for-performance program on patients with diabetes." Am J Manag Care 16(1): e11-19. 18. Chien, A. T., D. Eastman, et al. (2012). "Impact of a pay for performance program to improve diabetes care in the safety net." Prev Med 55 Suppl: S80-85. 19. Chien, A. T., Z. Li, et al. (2010). "Improving timely childhood immunizations through pay for performance in Medicaid-managed care." Health Serv Res 45(6 Pt 2): 1934-1947. 20. Clinical Practice Improvement Centre. (2010). Clinical practice improvement payment: User guide V2.0, pilot scheme—phase two. Brisbane, Australia: Queensland Health 21. Coleman T, Lewis S, Hubbard R, Smith C. (2007) Impact of contractual financial incentives on the ascertainment and management of smoking in primary care. Addiction; 102(5):803-808. 22. CORT, Vadodara. Report on assessment of ASHA/JSY scheme (Rajasthan) (2007). Commissioned by Ministry of Health & Family Welfare, Government of India. Draft. 23. Cupples ME, Byrne MC, Smith SM, et al. (2008). Secondary prevention of cardiovascular disease in different primary healthcare systems with and without pay-for-performance. Heart;94:1594e600. 24. Cutler TW, Palmieri J, Khalsa M, Stebbins M (2007). Evaluation of the relationship between a chronic disease care management program and California pay-for-performance diabetes care cholesterol measures in one medical group. Journal of Managed Care Pharmacy; 13(7):578-588. 25. Doran T, Kontopantelis E, Valderas JM, et al. (2011). Effect of financial incentives on incentivised and non-incentivised clinical activities: longitudinal analysis of data from the UK Quality and Outcomes Framework. *BMJ*.;342:d3590. 26. Eichler R, Auxila P, Antoine U, Desmangles B. (2007). Performance-based incentives for health: six years of results from supply. side programs in Haiti. CGD Working Paper #121.Washington, DC: Center for Global Development 27. Fagan PJ, Schuster AB, Boyd C, Marsteller JA, Griswold M, Murphy SM, et al. (2010). Chronic care improvement in primary care: evaluation of an integrated pay-for-performance and practice-based care coordination program among elderly patients with diabetes. Health Serv Res.;45:1763-82. 28. Fairbrother G, Hanson KL, Friedman S, Butts GC. (1999). The impact of physician bonuses, enhanced fees, and feedback on childhood immunization coverage rates. American Journal of Public Health; 89(2):171-175. 29. Fairbrother G, Siegel MJ, Friedman S, Kory PD, Butts GC. (2001). Impact of financial incentives on documented immunization rates in the inner city: results of a randomized controlled trial. Ambul Pediatr. ;1:206-12. [PMID: 11888402] 30. Felt-Lisk S, Gimm G, Peterson S. (2007). Making pay-for-performance work in Medicaid. Health Affairs; 26(4):W516-W527. 31. Friedman, N. L., Kokia, E., & Shemer, J. (2003). Health value added (HVA): Linking strategy, performance, and measurement in healthcare organizations. Israel Medical Association Journal, 5, 3-8. 32. Furth, R. (2006). Zambia Pilot Study of Performance-Based Incentives, USAID http://www.qaproject.org/news/PDFs/ZambiaPerformancePilotStudyInitiatives.pdf (visited June 2008) 33. Gavagan TF, Du H, Saver BG, Adams GJ, Graham DM, McCray R, et al. (2010). Effect of financial incentives on improvement in medical quality indicators for primary care. J Am Board Fam Med.;23:622-31. [PMID: 20823357] 34. Gilmore AS, Zhao YX, Kang N, Ryskina KL, Legorreta AP, Taira DA et al. (2007). Patient outcomes and evidence-based medicine in a preferred provider organization setting: A six-year evaluation of a physician pay-for-performance program. Health Services Research; 42(6):2140-2159. 35. Glickman SW, Ou FS, Delong ER, Roe MT, Lytle BL, Mulgund J et al. (2007). Pay for performance, quality of care, and outcomes in acute myocardial infarction. Jama-Journal of the American Medical Association; 297(21):2373-2380. 36. Grady KE, Lemkau JP, Lee NR, Caddell C. (1999). Enhancing mammography referral in primary care. Preventive Medicine; 26(6):791-800. 37. Greenberg MR, Weinstock M, Fenimore DG, Sierzega GM. (2008). Emergency department tobacco cessation program: staff participation and intervention success among patients. J Am Osteopath Assoc; 108(8):391-396. 38. Gross R, Elhaynay A, Friedman N, Buetow S. (2008). Pay-for-performance programs in P4P programs Israeli sick funds. J Health Organ Manag ; 22(1):23-35. 39. Grossbart, S. R. (2006). What’s the return? Assessing the effect of “pay-for-performance” initiatives on the quality of care delivery. Medical Care Research and Review*, 63,* 29S-48S. 40. Gulliford MC, Ashworth M, Robotham D, Mohiddin A. (2007). Achievement of metabolic targets for diabetes by English primary care practices under a new system of incentives. Diabetic Medicine 2007; 24(5):505-511. 41. Harries AD, Salaniponi FM, Nunn RR, Raviglione M. (2005). Performance-related allowances within the Malawi National Tuberculosis Control Programme. International Journal of Tuberculosis and Lung Disease; 9(2):138-144. 42. Hillman AL, Ripley K, Goldfarb N, Nuamah I, Weiner J, Lusk E. (1998). Physician financial incentives and feedback: failure to increase cancer screening in Medicaid managed care. Am J Public Health;88:1699-701. [PMID: 9807540] 43. Hillman AL, Ripley K, Goldfarb N, Weiner J, Nuamah I, Lusk E. (1999). The use of physician financial incentives and feedback to improve pediatric preventive care in Medicaid managed care. Pediatrics 1999; 104(4):931-935. 44. Hippisley-Cox, J., Vinogradova, Y., and Coupland, C. Final report for the Information Centre for Health and Social Care: time series analysis for 2001-2006 for selected clinical indicators from the QOF. [http://www.qresearch.org/Public_Documents/Time%20Series%Analysis%20for%20selected%20clinical.pdf](http://www.qresearch.org/Public_Documents/Time%20Series%25Analysis%20for%20selected%20clinical.pdf). 45. Jha, A. K., K. E. Joynt, et al. (2012). "The Long-Term Effect of Premier Pay for Performance on Patient Outcomes." New England Journal of Medicine 366(17): 1606-1615. 46. Kirschner, K., J. Braspenning, et al. (2013). "Assessment of a pay-for-performance program in primary care designed by target users." Fam Pract 30(2): 161-171. 47. Kontopantelis, E., D. Reeves, et al. (2012). "Recorded quality of primary care for patients with diabetes in England before and after the introduction of a financial incentive scheme: a longitudinal observational study." BMJ Quality & Safety. 48. Kruse, G. R., Y. Chang, et al. (2013). "Healthcare system effects of pay-for-performance for smoking status documentation." Am J Manag Care 19(7): 554-561. 49. Kouides RW, Bennett NM, Lewis B, Cappuccio JD, Barker WH, LaForce FM. (1998). Performance-based physician reimbursement and influenza immunization rates in the elderly. American Journal of Preventive Medicine; 14(2):89-95. 50. Kouides RW, Lewis B, Bennett NM, Bell KM, Barker WH, Black ER et al. (1993). A Performance-Based Incentive Program for Influenza Immunization in the Elderly. American Journal of Preventive Medicine; 9(4):250-255. 51. Kuo, R. N. C., Chung, K.-P., & Lai, M.-S. (2011). Effect of the pay-for-performance program for breast cancer care in Taiwan. American Journal of Managed Care, 17(5 Spec No.), e203-e211.Performance Management Program New Zealand 2006 52. Larsen DL, Cannon W, Towner S. (2003). Longitudinal assessment of a diabetes care management system in an integrated health network. J Manag Care Pharm; 9(6):552-558. 53. LeBaron CW, Mercer JT, Massoudi MS, Dini E, Stevenson J, Fischer WM et al. (1999). Changes in clinic vaccination coverage after institution of measurement and feedback in 4 states and 2 cities. Archives of Pediatrics & Adolescent Medicine; 153(8):879-886. 54. Lee, T.-T., Cheng, S.-H., Chen, C.-C., & Lai, M.-S. (2010). A pay-for-performance program for diabetes care in Taiwan: A preliminary assessment. American Journal of Managed Care, 16, 65-69. 55. Levin-Scherz J, DeVita N, Timbie J. Impact of pay-for-performance contracts and network registry on diabetes and asthma HEDIS (R) measures in an integrated delivery network. Medical Care Research and Review 2006; 63(1):14S-28S. 56. Li, J., Hurley, J., DeCicca, P., & Buckley, G. (2010). Physician response to pay-for-performance—Evidence from a natural experiment. Hamilton, Ontario, Canada: McMaster University. 57. Li, Y.-H., Tsai, W.-C., Khan, M., Yang, W.-T., Lee, T.-F., Wu, Y.-C., & Kung, P.-T. (2010).The effects of pay-for-performance on tuberculosis treatment in Taiwan. Health Policy and Planning, 25, 334-341. 58. Lindenauer PK, Remus D, Roman S, Rothberg MB, Benjamin EM, Ma A et al. (2007). Public reporting and pay for performance in hospital quality improvement. New England Journal of Medicine; 356(5):486-496. 59. Lynch M. (1995). Effect of Practice and Patient Population Characteristics on the Uptake of Childhood Immunizations. British Journal of General Practice; 45(393):205-208. 60. MacBride-Stewart SP, Elton R, Walley T. (2008). Do quality incentives change prescribing patterns in primary care? An observational study in Scotland. Family Practice; 25(1):27-32. 61. Magee GM, Hunter SJ, Cardwell CR, Savage G, Kee F, Murphy MC et al. (2010). Identifying additional patients with diabetic nephropathy using the UK primary care initiative. Diabet Med.; 27(12):1372-1378. 62. Mandel KE, Kotagal UR. (2007). Pay for performance alone cannot drive quality. Archives of Pediatrics & Adolescent Medicine; 161(7):650-655. 63. McGovern MP, Boroujerdi MA, Taylor MW, et al. (2008). The effect of the UK incentive-based contract on the management of patients with coronary heart disease in primary care. *Fam Pract*. ;25(1):33-39. 64. McMenamin SB, Schauffler HH, Shortell SM, et al. (2003). Support for smoking cessation interventions in physician organizations: results from a national study. Med Care;41:1396e406. 65. Millett C, Gray J, Saxena S, et al. (2003). Impact of a pay-for-performance incentive on support for smoking cessation and on smoking prevalence among people with diabetes. CMAJ;176:1705e10 66. Morrow RW, Gooding AD, Clark C. Improving physicians' preventive health care behavior through peer review and financial incentives. Arch Fam Med 1995; 4(2):165-169. 67. Norton EC. (1992). Incentive regulation of nursing homes. J Health Econ.;11: 105-28. 68. Oluwatowoju I, Abu E, Wild SH, Byrne CD. (2010). Improvements in glycaemic control and cholesterol concentrations associated with the Quality and Outcomes Framework: a regional 2-year audit of diabetes care in the UK. Diabet Med. ;27(3):354-359. 69. Peabody, J., R. Shimkhada, et al. (2011). "Financial incentives and measurement improved physicians' quality of care in the Philippines." Health Aff 30(4): 773-781. 70. Purdy S, Griffin T, Salisbury C, Sharp D. Emergency admissions for coronary heart disease: a cross-sectional study of general practice, population and hospital factors in England. Public Health. 2011;125(1):46-54. 71. Queensland Health. (2010). Clinical practice improvement payment. Retrieved from http://www.health.qld.gov.au/cpic/service_improve/cpip.asp 72. Rosenthal MB, Frank RG, et al. (2005). Early experience with payfor- performance: From concept to practice. JAMA; 294(14):1788–93. 73. Roski J, Jeddeloh R, An L, et al. (2003). The impact of financial incentives and a patient registry on preventive care quality: increasing provider adherence to evidence-based smoking cessation practice guidelines. Prev Med 2003;36:291e9. 74. Ryan AM. (2009). Effects of the Premier hospital quality incentive demonstration on Medicare patient mortality and cost. Health Services Research; 44(3):821-842. 75. Salize HJ, Merkel S, Reinhard I, et al. (2009). Cost-effective primary care-based strategies to improve smoking cessation: more value for money. Arch Intern Med;169:230e5 76. Schauffler HH, Brown C, Milstein A. (1999). Raising the bar: The use of performance guarantees by the Pacific Business Group on Health. Health Affairs; 18(2):134-142. 77. Scott A, Schurer S, Jensen PH, Sivey P (2009). The effects of an incentive program on quality of care in diabetes management. Health Economics , 18(9):1091-1108. 78. Serumaga B, Ross-Degnan D, Avery AJ, Elliott RA, Majumdar SR, Zhang F, et al. (2011). Effect of pay for performance on the management and outcomes of hypertension in the United Kingdom: interrupted time series study. BMJ. ; 342:d108. [PMID: 21266440] 79. Shen Y. (2003). Selection incentives in a performance-based contracting system. Health Serv Res.;38:535-52. 80. Simpson CR, Hannaford PC, Lefevre K, et al. (2006). Effect of the UK incentive-based contract on the management of patients with stroke in primary care. Stroke;37:2354e60. 81. Simpson CR, Hannaford PC, Ritchie LD, Sheikh A, Williams D. (2011). Impact of the pay-for-performance contract and the management of hypertension in Scottish primary care: a 6-year population-based repeated cross-sectional study. Br J Gen Pract.;61:e443-51. [PMID:21722469] 82. Simpson CR, Hippisley-Cox J, Sheikh A. (2010). Trends in the epidemiology of smoking recorded in UK general practice. Br J Gen Pract;60:e121e7. 83. Srirangalingam U, Sahathevan SK, Lasker SS, Chowdhury TA. (2006). Changing pattern of referral to a diabetes clinic following implementation of the new UK GP contract. British Journal of General Practice; 56(529):624-626. 84. Ssengooba, F., B. McPake, et al. (2012). "Why performance-based contracting failed in Uganda – An “open-box” evaluation of a complex health system intervention." Social Science & Medicine 75(2): 377-383. 85. St Jacques PJ, Patel N, Higgins MS. (2004). Improving anesthesiologist performance through profiling and incentives. J Clin Anesth 4;16:523-8. [PMID:15590256 86. Strong M, South G, Carlisle R. (2009). The UK Quality and Outcomes Framework pay-for-performance scheme and spirometry: rewarding quality or just quantity? A cross-sectional study in Rotherham, UK. BMC Health Serv Res; 9:108. 87. Sussman AJ, Fairchild DG, Coblyn J, Brennan TA.(2001). Primary care compensation at an academic medical center: A model for the mixed-payer environment. Academic Medicine; 76(7):693-699. 88. Sutton, M., S. Nikolova, et al. (2012). Reduced mortality with hospital pay for performance in England. N Engl J Med 367(19): 1821-1828. 89. Tahrani AA, McCarthy M, Godson J, et al. (2007). Diabetes care and the new GMS contract: the evidence for a whole county. Br J Gen Pract ;57:483e5. 90. Tsai, W.-C., Kung, P.-T., Khan, M., Campbell, C., Yang, W.-T., Lee, T.-F., & Li, Y.-H. (2010). Effects of pay-for-performance system on tuberculosis default cases control and treatment in Taiwan. Journal of Infection, 61, 235-243. 91. Twardella D, Brenner H. (2007). Effects of practitioner education, practitioner payment and reimbursement of patients' drug costs on smoking cessation in primary care: a cluster randomised trial. Tobacco Control; 16(1):15-21. 92. Vaghela P, Ashworth M, Schofield P, Gulliford MC. (2008). Population intermediate outcomes of diabetes under pay for performance incentives in England from 2004 to 2008. Diabetes Care. 93. Vergeer P, Chansa C. Payment for Performance (P4P) Evaluation: Zambia Country Report for Cordaid. KIT, Amsterdam. 94. Werner, R. M., R. T. Konetzka, et al. (2013). "The effect of pay-for-performance in nursing homes: evidence from state Medicaid programs." Health Serv Res 48(4): 1393-1414. 95. Yao H, Wei X, Liu J, Zhao J, Hu D, Walley JD. (2008). Evaluating the effects of providing financial incentives to tuberculosis patients and health providers in China. International Journal of Tuberculosis and Lung Disease; 12(10):1166-1172. 96. Young G, Meterko M, et al. (2007). Effects of paying physicians based on their relative performance for quality. Journal of General Internal Medicine ;22(6):872–6. |
| 1. Hamilton et al., 2013 2. Houle et al., 2012 3. Huang et al., 2013 4. Petersen et al., 2006, 5. Reda et al., 6. Scott et al., 2011 7. Van Herck P et al., 2010, 8. Witter et al., 2012 |  |

**Table S3. Search strategy output for economic theories to inform the P4P typology**

| Database | **PubMed, PsycINFO, EconLit,** |
| --- | --- |
| Host | http://ovidsp.tx.ovid.com/sp-3.13.1a/ovidweb.cgi |
| Date of search | January 2012-June 2014 last date searched: 26/6/14 |
| Years covered | 1990-2014 no date restrictions |
| Search Strategy | **You searched:**  ((behavioural economics or behavioural theories or incentive theories or economic theories) and incentive).mp. [mp=hw, ab, ti, ct, sh, tn, ot, dm, mf, dv, kw, nm, kf, px, rx, an, ui, tc, id, tm]  ***-****Search terms used:*   - behavioural - behavioural economics - behavioural theories - economic - economic theories - economics - incentive - incentive theories - theories |
| Language restrictions | None |
| Number of citations | 170 |

**Table S4. Application of the typology on selected identified P4P schemes**

| **Program** | **Perceived risk** | **Incentive size** | **Who receives the incentive** | **Fines or bonuses** |
| --- | --- | --- | --- | --- |
| Advancing Quality  United kingdom  2008 | High risk  Annually (long time lag)  Mostly within Physicians control (2 final outcomes and 26 processes)  Relative measure | Small  2-4% | Group | Bonuses |
| Clalit  Israel, 1998 | Low risk  Annually (long time lag)  Mostly within Physicians control (10 processes and 8 intermediate outcomes)  Absolute measure | Large Dependent on budget savings | Groups | Bonuses |
|  |  |  |  |  |
|  |  |  |  |  |
|  |  |  |  |  |
| Clinical Practice Improvement Pay (CPIP)  Australia, Queensland (started 2008) | Low risk  Semi-annually (long time lag)  Within physicians control (12 structures and 7 processes)  Absolute measure | Large  8-10% | Group | Bonuses |
| MACCABI  Israel  2001 | High risk  Annually (long time lag)  Mostly within Physicians control (12 processes and 5 intermediate outcomes)  Relative measure | Most likely large  Size not reported | Group | Bonus |
| National Health Insurance P4P (NHI-P4P)  Taiwan  2004 | High risk  Monthly and annually  12 structures, 3 final outcomes, and 2 intermediate outcomes  Absolute and relative measures | Large  Up to 20% | Individuals and groups | Bonuses |
| Primary care P4P (PC-P4P)  Netherlands | High risk  Annually (long time lag)  Within physicians control (31 processes)  Relative measures | Large  8-10% | Individual and groups | Bonuses |
| Primary Care Renewal Models (PCRM)  Canada Ontario  Started 2007 | Low risk  Annually Within physicians control (12 processes)  Absolute measure | Small  2-4% | Individual and groups | Bonuses |
| Physician Integrated Network (PIN)  Canada Manitoba  2004 | Low risk  Immediately after performance measure (short time lag)  Within physicians control (only processes)  Absolute | Maximum payment unknown but likely large | Groups | Bonuses |
| Practice Incentive Program (PIP)  Australia 1998 | Low risk  Quarterly, semi-annually and annually, Within physicians control (only structures and processes)  Absolute measure | Size not reported relative to income but likely small | Group | Bonuses |
| Quality and Outcomes Framework (QOF) | Low risk  Annually (long time lag)  Mostly within physicians control (85% processes)  Absolute measure | Large  Up to 30-40% | Group | Bonuses |
| Western New York Physician Incentive Program (WNY-PIP)  USA | Low risk  Annually (long time lag)  Mostly process: 6 Process and 3 outcomes  Intermediate outcome  Absolute measure | Size of varied from $3,000 till $12,000  large | Individuals | Bonus |
|  |  |  |  |  |
| Kouides et al., 1998  Rochester, New York, USA | Low risk  Annually (long time lag)  Process  Absolute measure | Size  ‘Modest’ for just one process? | Group | Bonus |
| Ashworth et al., 2004  UK 2004 | Low risk  Annually (long time lag)  Process/structure  Absolute measure | up to £5000 per GP  (large)  Up to 5% | Groups but money trickled down to individuals | Bonus |
| Cattaneo et al., 2001  Italy  1998-1999 | Low risk  Yearly (long time lag)  Process  Absolute measure | Small 0.5% of annual revenue deducted | Groups | Fines |
| Fairbrother et al., 1999  New York  12 months | Low risk  Annually (long time lag) Process  Absolute measure | $1000  Large | Individuals | Bonus plus feedback |
| Fairbrother et al., 2001  USA  16 months | Low risk  One off payment after 16 months (long time lag)  Process  Absolute measure | 1000 usd | Individual | Bonus |
| Grady et al., 1997  USA | Low risk  Quarterly payments (short time lag)  Process  Absolute  Measure | Token  Small?  , i.e., $50 for a 50% referral rate.  Small up to 1% | Groups | Bonus with education |
| Hillman et al., 1998 | Low risk  Every 6 months (long time lag)  Process  Absolute measure | Large Up to 20% of capitation fees | Individuals and groups | Bonus and feedback 18 months: no effect |
| LeBaron et al., 1999  USA | Not enough information reported on the costs and nature of incentives |  |  | Bonuses |
| Rooski et al., 2003  USA | Low risk  3 month time lag in payment  Process  Absolute measure | Size: up to $10,000 not reported relative to practice budget/income  Most likely large. | Groups | Bonuses |
| Ritchie et al., 1991  Scotland: UK | Low risk Quarterly payments (short time lag)  Process  Absolute measure | Not enough information reported on size | Groups  Clinical practices | Bonuses |
| Hillman et al., 1999  USA | Low risk  Process  Absolute and relative really  Payment frequency: every 6 months | Bonuses based on total compliance score for quality indicators; full and partial bonuses  Average bonus, $2,000 (range, $772 to $4682) | Payments to provider groups | Bonuses  Feedback |
| Hillman et al., 1998  USA | Low risk  Payment frequency: every 6 months (long time lag)  Process  Absolute measure | $1260  Large: up to20% | Provider group | Bonuses |
| Chien et al., 2012 Hudson Health Plan's P4P program in New York | High risk  Both process and outcomes  Yearly  Absolute | 300$ per patient | Groups | Bonuses |
| Harries et al., 2005  Malawi National Tuberculosis Control Programme  (four year program/0 | Low risk  6month (short time lag)  process  absolute measure | Size: up to 100% of usual reimbursement | Individual physicians | Bonuses |
| Gavagan, et al., 2010  USA | Low risk  Annually (long time lag)  Processes  Absolute Measure | Small  approximately 3% to 4% of a  provider’s total salary | Individual physicians | Bonuses |
| An et al., 2008  USA | Low risk  Annual (long time lag)  Process  Absolute measure | Small  5000$ onetime payment at the end of the programme | Groups | Bonuses |
| Glickman et al.,2007  USA  CMS  Premier program | High risk  Yearly (long time lag)  Process and outcomes  Relative | Small 2% | Groups (hospitals) | Bonuses |
| [Mandel](http://www.ncbi.nlm.nih.gov/pubmed?term=Mandel%20KE%5BAuthor%5D&cauthor=true&cauthor_uid=17606827) et al., 2007  Cincinnati  USA | Can’t tell: not enough information reported  Process | Large  7% fee schedule increase | Practices (groups) | Bonuses |
| Greenberg et al., 2008 | Low risk  Payment every three months (short time lag)  Process | Not enough informtion reported | Individuals | Bonuses |
| Levin et al., 2006  USA | Low risk  Paid monthly (short time lag)  Process  Relative measure | Up to 20% of budget/salary | Groups | Bonuses |
| Christensen et al., 2000  USA | Low risk  Timing of payment not reported  Process  Absolute measure | $4 for cognitive services | Provider group | Bonuses |
| Fagan et al., 2010 | Low risk  Timing of payment not reported  Process and structures  Absolute measure | Large  Up 20% | Groups | Bonuses |
| Yao H et al., 2008  China | Not enough information reported  Process | $31 694 for spreading TB knowledge in villages | Doctors  Individuals | Bonuses |
| Jha et al., 2012  CMS | High risk  Yearly (long time lag)  Process and outcomes  Relative measure | 2% | Groups  hospitals | Bonuses |
| Basinga et al., 2011  Rwanda | Low risk  Monthly and quarterly payments (short time lag)  Processes  Absolute measure | Large 22-38% of usual budget and salary | Individuals and groups | Bonuses |
| Chien et al., 2010  USA | Low risk  Timing of payment not reported  Process  Absolute measure | Large | Individuals | Bonuses |
| Lynch et al.,1995 | Annually  Paid quarterly  Absolute (tournament) it would between 70% and 89%; rates below 70% do not qualify for these payments.  Low risk |  | Paid to GP practices  Groups | Bonuses |
| Sussman et al., 2000  Boston, Massachusetts  USA | Low risk  Yearly (long time lag)  Process  Absolute measure | Large Size: up to 10% of salary | Bonuses | Groups |
| Norton et al.,1992 | High risk  Can’t tell  Timing of payment not reported: yearly Outcomes  Absolute measure | Large $126 to $370 | Groups | Bonuses |
| Shen et al., 2003  Maine, USA | Low risk  Annual payment (long time lag)  Process  Absolute measure | Not enough reported about size | Groups | Bonuses |
| Werner et al., 2012  CMS  USA | High risk  Yearly (long time lag)  Process and outcomes  Relative measure  Yearly  HIGH RISK | Small 2% | Groups | BONUSES |
| Canavan A. and Swai G. (2008)  Tanzania | Low risk  Payment every 6 months (long time lag)  Processes  Absolute measure | Large 5-10% of hospital budget and clinicians salary | Individuals and groups | Bonuses |
|  |  |  |  |  |
| Sulku, 2011  Turkey | Low risk  Monthly payments (short time lag)  Process and outcomes  Absolute measure | Large  Up to 80% of budget and salary | Individuals and groups | Bonuses |
|  |  |  |  |  |
| Vergeer and Chansa, 2008.  Zambia | Low risk  Absolute measure  Quarterly payments (short time lag)/Processes | Up to 100% of salary | Individuals and groups | Bonuses |
| Cutler et al., 2007  USA (California P4P) | High risk  Annual payments (long time lag)  Processes and intermediate outcomes  Relative measure | Large  Up to 5% of budget | Groups | Bonuses |
| Ssengooba et al., 2012.  Uganda | Low risk  6monthly payment (long time lag)  Process  Absolute measure | Large up to 11% of hospital budget | Groups | Bonuses |
| Gilmore et al., 2007  Hawaii Medical Services Association | High risk  Annual (long time lag)  Relative  Outcomes | Large  Up to 7% of salary | Individuals | Bonuses |
| Young et al., 2007 | High risk  Annual (long time lag)  Processes  Relative measure | Large 5% of physician fees was at risk | Individuals | Fines |
| Schauffler et al., 1999  California  USA | Low risk  Annual (long time lag)  Processes  Absolute measure | Small  up to 2% of premiums at risk | Groups | Fines |
| Twardella and Brenner, 2007 | High risk  Annual (long time line)  Outcome  Absolute measure | Unclear | Individuals | Bonuses |
| Kouides et al., 1993 | Low risk  Annual payment (long time lag)  Processes  Absolute | Unclear | Individuals | Bonuses |
| St Jacques et al., 2004 | low risk  Monthly payment  Processes  Relative | Large  Up to 500 dollars per month | Individuals | Bonuses |
|  |  |  |  |  |
|  |  |  |  |  |
| Salize et al., 2009 | High risk  Payment after a year  Outcomes (quit rate)  Absolute | financial incentive of (€130) | Individuals | Bonuses |
| McMenamin et al., 2003 | Low risk  Process  Absolute | Not reported | Groups | Bonuses |
| Chee et al, 2007  GAVI Incentives for national governments | Low risk  Time lag not clear  Processes  Absolute measure | Up to 15% increased immunization funding  (large) | National government: institutions/groups | Bonus |
| Eichler et al., 2007  Haiti: RBF for NGO | Low risk  Quarterly payments  Processes  Absolute measure | Up to 15% of previous budget of NGO  (large) | NGOs: groups/institutions | Bonus |
| CORT 2007 | Low risk  Payment every three months  Processes  Absolute measure | $4.94 to $34.58  (large as per Indian standards) | health professionals (ASHA’s) (individuals) | Bonuses |
| Chen et al., 2010 | Low risk  Annually  Processes  Absolute | Large  Up to 7.5% of salary | Individuals | Bonuses |
| Armour et al., 2004 | Low risk  End of the year payments  Processes  Absolute measure | Size unknown | Individuals | Bonuses |
| Bardach et al., 2014 | Low risk  Unclear timing of payment  Processes  Absolute measure | large | Groups | bonuses |
| Greene et al., 2004 | High  Yearly  Process and outcomes  Relative | Large  Up to 20% of capitation fees | Individuals | Withholds  Fines |
| Bischoff et al, 2012 | Low risk  Payment after a year  Processes  Absolute | Unclear | Groups | Bonuses |
|  |  |  |  |  |
|  |  |  |  |  |
| Boland et al., 2010 | Low risk  Payment at 6 months intervals  Processes  Absolute measure | Up to $5000 annually  Large | Individuals | Bonuses |
|  |  |  |  |  |
| Kruse et al., 2013 | Low risk  Payment after 2 years  Processes  Absolute | Large  Approximately 5% | Groups | Bonuses |
| Peabody et al., 2011 | Low risk  Payment date no known  Process  Absolute | Large approximately 5% of clinicians salary | Groups and individuals | Bonuses |

**Table S5 P4P studies used in testing the inter-rater reliability of the P4P typology**

| 1. An, L.C., et al., *A randomized trial of a pay-for-performance program targeting clinician referral to a state tobacco quitline.* Arch Intern Med, 2008. 168(18): p. 1993-9.  2. Ashworth, M., et al., *How are primary care organizations using financial incentives to influence prescribing?* Journal of Public Health, 2004. 26(1): p. 48-51.  3. Basinga, P., et al., *Effect on maternal and child health services in Rwanda of payment to primary health-care providers for performance: an impact evaluation.* The Lancet, 2011. 377(9775): p. 1421-1428.  4. Beaulieu, N.D. and D.R. Horrigan, *Putting smart money to work for quality improvement.* Health Serv Res, 2005. 40(5 Pt 1): p. 1318-34.  5. Catteneo, A., B. Giulio, and S. Giorgio, *Breasfeeding by objectives.* European Journal of Public Health, 2001. 11: p. 397-401.  6. Fairbrother, G., et al., *The impact of physician bonuses, enhanced fees, and feedback on childhood immunization coverage rates.* American Journal of Public Health, 1999. 89(2): p. 171-175.  7. Fairbrother, G., Hanson, K.L., Butts, G.C., Friedman, S., *Comparison of preventive care in medicaid managed care and medicaid fee for service in institutions and private practices* Ambulatory Peadiatrics, 2001. 1: p. 294-301.  8. Harries, A.D., et al., *Performance-related allowances within the Malawi National Tuberculosis Control Programme.* The International Journal of Tuberculosis and Lung Disease, 2005. 9(2): p. 138-144.  9. Jha, A.K., et al., *The Long-Term Effect of Premier Pay for Performance on Patient Outcomes.* New England Journal of Medicine, 2012. 366(17): p. 1606-1615.  10. Kirschner, K., et al., *Assessment of a pay-for-performance program in primary care designed by target users.* Fam Pract, 2013. 30(2): p. 161-71.  11. Kouides, R.W., et al., *Performance-based physician reimbursement and influenza immunization rates in the elderly.* American Journal of Preventive Medicine, 1998. 14(2): p. 89-95.  12. Li, Y.H., et al., *The effects of pay-for-performance on tuberculosis treatment in Taiwan.* Health Policy Plan, 2010. 25(4): p. 334-41.  13. Gavagan, T.F., et al., *Effect of Financial Incentives on Improvement in Medical Quality Indicators for Primary Care.* J Am Board Fam Med, 2010. 23: p. 622– 631.  14. Roski, J., et al., *The impact of financial incentives and a patient registry on preventive care quality: increasing provider adherence to evidence-based smoking cessation practice guidelines☆☆Surveys available upon request from corresponding author.* Prev Med, 2003. 36(3): p. 291-299.  15. Ssengooba, F., B. McPake, and N. Palmer, *Why performance-based contracting failed in Uganda – An “open-box” evaluation of a complex health system intervention.* Social Science & Medicine, 2012. 75(2): p. 377-383.  16. Sutton, M., et al., *Reduced mortality with hospital pay for performance in England.* N Engl J Med, 2012. 367(19): p. 1821-8.  17. Werner, R.M., R.T. Konetzka, and D. Polsky, *The effect of pay-for-performance in nursing homes: evidence from state Medicaid programs.* Health Serv Res, 2013. 48(4): p. 1393-414. |
| --- |

**Table S6 Rater population**

| The rater population consisted of five PhD students, four Master’s students, and three health service researchers (with a Master’s degree being their highest qualification). Four of the raters had between zero to one year of research experience, seven raters had between two to four years of research experience, and one rater had over five years of research experience. Three of the raters had previous research experience in or were currently working on P4P schemes in healthcare.  A training manual was developed to train the volunteer raters. This included clear and concise decision rules (with examples where needed) to accompany the guidance for applying the tool to the P4P schemes. Volunteer raters were trained face to face or over skype on how to use the typology to categorize P4P schemes. The raters were asked to rate the studies independently. |
| --- |

**Table S7 Sources of disagreement**

| Sources of disagreements between the raters were random and not specific to any particular rater. The sources of disagreement in the third and fourth item (size of incentive and perceived risk of not earning the incentive) reflected subjective rater judgement.  Table 8 illustrates raters’ responses to judging the size of incentive in a P4P study, which according to the typology guideline should be considered small if less than 5% of usual salary or budget and large if 5% or more than usual salary or budget. Item 4 (‘risk’) consist of three design variables (timing of payment, domain of performance, and performance measure), therefore, there is higher likelihood of disagreement between the raters because differences in judgement of just one of the design features led to different categorisations regarding the fourth item. Table 9 shows examples of sources of disagreement on item 4 (risk). Both raters agreed on categories of domain of performance and performance measure, but one of the raters was unclear about the timing of payment and had indicated that he/she judged subjectively (the typology states that timing of payment should be considered short if payment is made anytime within four months of measurement of performance, while payments made after 4 months is considered long). The lack of clarity as pointed out by the raters reflects lack of clarity and structure in reporting design features in the P4P papers, which supports our argument for the need for a uniform reporting template and the adoption of our developed tool-the Healthcare Incentives Reporting Framework (HISReF). |
| --- |

**Table S8 An example of source of disagreement between raters (risk)**

| **Quote/extract from study (Werner et al. 2011)**[**^67^**](#_ENREF_67) | | |
| --- | --- | --- |
| **Rater 1** | **Time lag: short or long** | **Perceived risk of not earning the incentive: high or low risk** |
|  | **Domain of measurement: within the clinicians control or out of clinicians control** |  |
|  | **Performance measure: absolute or relative measure** |  |
|  | Unclear**:** The study does not specify the time lag between performance measure confirmation and payouts. It might have been a short time lag | Low risk |
|  | Processes (within clinicians control); For two of the three clinical conditions we studied, Medicare’s composite measures are based exclusively on process measures. |  |
|  | Partially relative; Two additional payment incentives were introduced in the fourth year (fiscal year 2007). Hospitals that attained a target performance level (defined as median performance two years previously) received an incentive. In addition, of the hospitals attaining that level, those that were in the top 20 percent in terms of improvement received another incentive. |  |
| **Rater 2** | Long time lag: The first two years of the demonstration project (fiscal years 2004 and 2005), financial bonuses were distributed to the top 20 percent of hospitals. | High risk |
|  | Processes (within clinicians control): Participating hospitals received higher payments for treating medicare patients with certain condition- acute myocardial infarction, heart failure, pneumonia, coronary artery bypass graft and knee and hip replacements. |  |
|  | Relative: Two additional payment incentives were introduced in the fourth year (fiscal year 2007). Hospitals that attained a target performance level (defined as median performance two years previously) received an incentive. In addition, of the hospitals attaining that level, those that were in the top 20 percent in terms of improvement received another incentive |  |
